# Bibliographical Notices

**Published:** 1856-03

**Authors:** 


### BIBLIOGRAPHICAL NOTICES.

*The Diseases and Functional Disorders of the Stomach.* By GEORGE BUDD, M. D., F. R. S., Professor of Medicine in King's College, London, &c. 8vo. pp. 252. Philadelphia: 1856. Blanchard & Lea.

*The Diseases and Functional Disorders of the Stomach.* By GEORGE BUDD, M. D., F. R. S., Professor of Medicine in King's College, London, &c. 8vo. pp. 252. New York: S. S. & W. Wood.

In the publication of this excellent work, Dr. Budd has given to the profession a very appropriate and acceptable companion to his former treatise on Diseases of the Liver. A careful examination of its contents has convinced us that it will compare favorably with that treatise, whose merits are already so well known, and add materially to the reputation of its author.

The volume comprises a series of lectures, originally published, from time to time, in some of the British medical journals. The remarks on self-digestion of the stomach after death, and on simple ulcer, were delivered as the Croonian lectures, at the College of Physicians. They are now republished, as the author informs us, with such additions and corrections as his subsequent experience has suggested.

Passing over the introductory comments upon the many difficulties attending the study of gastric diseases, the attention of the reader is at once arrested by the admirable remarks upon post-mortem digestion of the stomach,—a phenomenon interesting alike to the physiologist, the pathologist and the medical jurist. In alluding to the somewhat different results of the observations and experiments of Hunter, Spallanzani and Louis, and the more recent investigations of Mr. Wilkinson King, upon the solvent powers of the gastric juice, Dr. B. says:—

"No one seems to have observed this change [softening and perforation of the stomach] so frequently as Hunter; but the truthfulness of Hunter's mind, and the simplicity and candor of his statements, prevent us from supposing that in this respect he was guilty of any exaggeration. The fact is, that the solvent powers of the gastric juice require a certain temperature, and increase as the temperature rises from the lowest point at which they act at all, to the temperature of the blood, —the temperature at which they act in the living body. Hunter's observations were probably made during a hot summer, when, by reason of the high temperature, softening or digestion of the stomach after death was unusually frequent. During the past summer, which was a very hot one, my attention was carefully drawn to this subject, and from the middle of May to the middle of August, I carefully examined the stomach in all the bodies that were opened in the King's College Hospital. In several instances the mucous membrane in the great end of the stomach was completely destroyed, and in a very large proportion, it had been clearly acted on, more or less by the gastric juice. I renewed my observations in October, but the change, at least in a striking degree, was then much less frequent." (p. 23.)

The different circumstances under which softening of the stomach occurs, and the inferences to be drawn from them, are detailed in a concise and practical manner :—

"Cruveilhier has distinguished two kinds of softening of the stomach, both occurring principally in its big end, and has called them the *pulpy* softening, and the *gelatinous* softening, from the appearance respectively presented by the softened tissues. The pulpy softening he supposes to occur after death, from the action of the gastric juice ; the gelatinous softening during life, from a peculiar morbid process. Rokitansky, distinguishes three varieties of softening in this portion of the stomach, according to the color of the softened tissues ; and two of these he supposes to occur during life as the effect of disease.

"An attentive examination of these so-called affected varieties, leaves no doubt in my mind that they are all produced after death, and by the same agent, namely, the gastric juice ; and that the differences of transparency and color in the softened tissues, to which so much importance has been attached, result mainly from variations in the quantity of blood in these tissues at the time of death."

Dr. B. considers the following characters as sufficient, in most cases, to distinguish post-mortem digestion of the stomach, even in its slighter degrees, from all the other changes to which that organ is liable.

"1st. A softening of the mucous membrane, usually over a considerable space in the great end of the stomach, and *along the edges of the folds*, extending from this towards the pyloric end ; parts which, for reasons I have already assigned, are most exposed to the action of the dissolving agent.

"2d. A blackening of the blood in the tissues so acted upon, giving various shades of brown to the softened tissues when much blood was contained in them at the time of death.

"A third character of the change is, that the softened or digested tissues have an acid reaction ; and that they putrify less readily than other parts, in consequence of the antiseptic properties of the gastric juice."

Gastric softening takes place in simple ulcer of the stomach; in phthisis, as well described by Louis, who, however, appears not to have been aware of its true nature; in inflammatory diseases of the brain; in typhoid and other fevers; in peritonitis and uterine cancer, and in other abdominal diseases which lead to secondary functional disorders of the stomach.

Lecture III is a practical dissertation upon the causes, conditions and effects of congestion of the stomach. The influence of organic visceral disease in producing turgescence of the gastric vessels, by impeding the free passage of blood through the liver or chest, is clearly illustrated. Another and perhaps more interesting cause of this disorder, is to be found in certain abnormal conditions of the blood, which conditions are dependent upon a change in the relative proportions of the healthy constituents of that fluid, or upon the presence of some foreign matters in it, by which its consistence is altered, and its regular and orderly propulsion thus obstructed. Examples of this latter cause we find in yellow fever, Asiatic cholera and other malignant affections.

Dr. Budd next proceeds to treat of Inflammation of the Stomach,—a malady whose importance is as great as our knowledge concerning it is vague and uncertain. As the disease seldom proves fatal, opportunities of examining the inflamed tissues rarely occur. The morbid secretions, too, even when vomited freely, are very unreliable guides in arriving at an exact diagnosis, since they are generally mixed with other secretions, and chemically acted upon by the gastric juice; while if they are passed down into the duodenum, instead of being cast up into the œsophagus, they necessarily suffer a change from intermixture with the bile and pancreatic juice. For these reasons it will be seen that gastritis presents more obstacles to the exact and satisfactory elucidation of its various grades, than most of the phlegmasia. In the volume under consideration, the disease is treated of under the following heads: 1st. Inflammation excited by undigested food, or alcoholic drinks; 2d. Inflammation caused by more powerful mechanical or chemical irritants; 3d. Inflammation resulting from defective nutriment or from the presence of noxious matter in the blood.

In the 6th and 7th Lectures, Perforating and Superficial Ulcers are carefully discussed. Lecture VIII is devoted to the consideration of Cancer of the Stomach.

"The *symptoms* of cancer of the stomach have seldom any characters that are peculiar or especially significant, until the disease has existed a considerable time.

"The first complaint is generally of a dull pain, or a sense of uneasiness at the pit of the stomach, which comes on gradually, without fever or other illness, and is always aggravated by taking food. This pain or uneasiness is attended with impairment of appetite. and with flatulence, heartburn, and other results of disordered digestion.

"As the disease makes progress, the disorder of digestion increases. The patient usually loses all relish for food, is troubled with occasional eructation of a sour fluid, occurring at various hours of the day, and now and then, soon after meals, brings up, in the same manner, small quantities of food. He loses flesh, and in most cases becomes much depressed in spirits. Still he is free from fever; the pulse is no quicker than it should be; the tongue is clean or only slightly furred; and there is no unnatural heat of skin, and no thirst.

"The uneasiness at the epigastrium and the disorder of digestion continue, but the symptoms vary in some degree according to the seat and extent of the disease. When the cancer involves the body of the stomach and the orifices are unobstructed, vomiting, if it occurs at all occurs soon after meals, and what is brought up at a time is moderate in quantity, and consists of the secretions recently swallowed.

"When, as happens much more frequently, the disease is confined to the pyloric end of the stomach, the pyloric orifice usually becomes obstructed or narrowed, or the passage of the food through it is impeded by the restrained action of the muscular fibres. In such cases vomiting is a necessary result, but it takes place later after meals than in the former class of cases, more is brought up at a time, and the matters vomited are very sour.

"Several conditions,—undue retention of the food, a scanty supply of gastric juice, and the presence of unhealthy and decomposing secretions furnished by the cancerous surface,—conspire to promote in the contents of the stomach some unnatural fermentation, which leads to the formation of enormous quantities of acid, and often to an abundant evolution of gas. The acid frets the stomach, and much additional suffering results from its flatulent distension. Occasionally, and especially when a cancerous *ulcer* exists in the stomach, the food unduly detained there undergoes the butyric fermentation or common putrefactive changes, and thus gives rise to fetid eructations and the other symptoms of 'surfeit,' which, from their infrequency in other chronic diseases of the stomach, are often important evidence of the existence of cancer. In many cases, perhaps in all those in which the body of the stomach remains sound, the obstruction of the pyloric orifice leads, after a time, to great enlargement of the stomach. When this has taken place, vomiting occurs more rarely

—at the end of each day, of every two or three days, or at still longer intervals—and a great quantity is brought up at once; the matter ejected comprising the accumulated secretions of the stomach, and all the food not capable of absorption in the stomach that had been eaten since the last vomiting.

"When, on the other hand, the disease involves the *cardiac* orifice, the entrance of food into the stomach is impeded. The act of swallowing causes pain, or a sense of uneasiness, at the epigastrium, passing through to the back; and very commonly a small portion of what is swallowed lodges in the lower end of the œsophagus, and is immediately or speedily brought up again by an effort of eructation rather than of vomiting. The patient himself is aware that the food does not readily enter the stomach.

"In all cases where much is rejected by vomiting, and but little consequently passes into the duodenum, the bowels become confined; but where the pylorus is not obstructed, the costiveness is interrupted now and then by a transient diarrhœa, produced by the irritating matters which pass from the stomach into the bowel."

Occasionally with the glairy mucus thrown up, are mixed brown or black flakes, consisting of altered blood; and frequently after ulceration, there is an abundant hemorrhage. Generally, however, by this time, we are able to detect the tumor formed by the cancerous mass, particularly if the pyloric end of the stomach, which is the most common seat of the disease, be involved. Cases are not very uncommon, though, where some of the most characteristic symptoms are wanting. Thus there may be little pain, or the tumor may be small and at the back of the stomach, and the vomiting may be so slight as hardly to attract attention. The absence of this latter symptom is owing to the fact that neither of the orifices are affected, and that the disease involves only a small portion of the hind part of the stomach.

Cancer of the stomach rarely occurs before the age of thirty-five.

The object of the treatment should be to lessen the sufferings of the patient; by regulating his diet and reducing the food to an impalpable pulp, the pain is much lessened.

Where much mucus is secreted, bismuth has proved to be of much benefit. Where there is an excess of acid, relief is obtained by lime water, magnesia, and other mild antacids. If fœtid eructations be present, creasote, or finely powdered wood charcoal may often be given with advantage.

These remedies, with an occasional pill of aloes or colocynth, to relieve constipation, and the different preparations of opium, conium and belladonna, to mitigate pain and procure sleep, are the principal means in our power for alleviating suffering or prolonging life in this fell disease.

In the next three Lectures, the reader's attention is directed to the Sympathetic Disorders of the Stomach originating from irritation in other parts of the economy; slow and imperfect digestion produced by deficient secretion of gastric juice; and fermentation in the contents of the stomach, with development of sarcinæ. The causes, varieties, peculiar symptoms and complications of that hydra-headed disease, indigestion, constitute the subject matter of the succeeding three Lectures; while the remaining two treat of the therapeutic value of the various remedies employed in the treatment of gastric affections.

This brief notice will suffice to give our readers a general idea of the character and contents of the work before us. Our limited space prevents a more extended examination.

---

*Report on Insanity and Idiocy in Massachusetts. By the Commission on Lunacy, under resolve of the Legislature of* 1854.

The ratio which the insane and idiotic bear to the entire population has engaged the attention of most civilized nations; but we question if the most accurate estimate that has hitherto been formed, can claim to be more than very loosely approximative. Certain it is that in the last United States census, little over two-thirds of the insane and idiotic were returned by the marshals; and in Great Britain the estimates only included paupers, lunatics and those confined in asylums, taking no account of that "considerable class of insane persons of all ranks of life, under the care of guardians and relations;" while a careful analysis of the returns of the agents of the French government suggest strong doubts as to their accuracy.

Fortunately, the above commission embraced men who fully understood the errors of former inquirers, and had the ability to devise the means and perseverance to accomplish for Massachusetts a most accurate enumeration of the insane and idiotic within her borders. The plan pursued by the Commissioners was as follows: "They considered that there are very few families that are not within the personal knowledge of some practitioner of medicine, and that therefore the whole commonwealth is, in detail, under the eye of the medical profession; and that they, knowing the domestic condition of the whole people, were of course acquainted with all those whose minds were disordered or defecttive." Accordingly, the commission addressed a lithograph letter to "every physician in the State, asking each one to give information relative to the person and condition of all the lunatics and idiots within his own knowledge. They asked for the name, sex, color, age, country of birth, whether single, married or widowed, whether lunatic or idiot; present and usual condition; whether mild, manageable, troublesome, excitable, furious or dangerous; whether subject for a hospital or not; length of disease; if periodical, the number of attacks; whether curable or not; whether the remedial influences of any hospital had ever been tried for restoration; where resident, if not in the town of the reporter; and whether State or town paupers, or independent." Similar letters were also sent "to the superintendents of the Lunatic Hospitals in Worcester, Taunton, Somerville and Boston; to the officers of the county receptacles for the insane in Cambrige and Ipswich, and personal inquiry was made of masters of all the houses of correction and jails in the State, and of the proprietors of all private houses or establishments devoted to the care of the insane; asking each to make a similar return of the lunatics and idiots under his care. And, in order to complete this survey, letters were sent to officers of all the hospitals in the Northern and Middle and some of the Southern States, asking them to make returns of all the insane patients belonging to Massachusetts, who were entrusted to their care." Besides these, the commission received assistance from the hands of gentlemen out of the profession, clergymen, overseers of the poor, selectmen in several towns, where aid was wanted. "By this means, the commission have ascertained, that, in 1854, the State of Massachusetts contained 2632 lunatics and 1087 idiots;" whereas, "in 1848, a committee of the Legislature, appointed to 'consider the whole subject connected with insanity within the commonwealth,' ascertained and reported the number of insane in this State to be fifteen hundred and twelve;" while in

1850, the marshals engaged in taking the national census, reported only 1680 lunatics and 791 idiots.

Of the lunatics returned by the present commission, 1522 were paupers, 1110 "were supported by their own property, or by their friends,"—2007 were natives, and 625 were foreigners,—435 were curable, 2018 incurable, 197 not stated. Of the idiots, 670 are supported by friends, 417 are paupers—1043 are natives, 44 foreigners.

The close relationship between insanity and poverty, is forcibly illustrated in this report, showing, as it does, that the pauper class furnishes in proportion to its members 64 times as many cases of insanity as the independent class; while the number of incurables among the former, amounts to eighty-six per cent., whereas, among this latter, only seventy-five per cent .are returned as beyond the hope of recovery.

It is also worthy of notice, though easily accounted for, that foreign lunatics bear a larger ratio to the sane population of their own class, than the native lunatics; thus "the native insane were one in 445 of the total native population, and the foreign insane were one in 368 of the whole number of aliens in the State." The vast majority of the foreign population of Massachusetts is Irish; but as it is undeniable that insanity is not more prevalent in Ireland than in the United States, we must attribute its greater development among them in their adopted country to causes acting subsequent to their expatriation. These causes seem to act very unequally on the two sexes of immigrants, as among the males the ratio which the insane bear to the sane population is one in 435; and among the females it is as high as one in 326. Among the natives the proportion to the population is nearly equal—one in every 444 males, and one in every 443 females.

The curability of insanity, if placed under suitable treatment within the first year of its duration, is stated by the commissioners to be as high as 75 to 90 per cent.; but if a second year is allowed to elapse before restorative measures are resorted to, the cures would be less than one half that proportion. After the fifth year has passed, almost all hope of recovery may be abandoned. There can be no doubt of the advantages of an early resort to treatment, but we question much, if, under the most favorable circumstances, the ratio of cures in fully developed insanity ever reaches 90 per cent. When the reported "cures" reach so high a figure, we cannot but fear that the temporary recoveries of more than one recurrent case are reported as permanent "cures," maugre the fact that after a few years "yeoman's service" of this kind, the poor patient may sink into hopeless dementia.

As regards the idiots of Massachusetts, we have only space for the "noticeable fact, that a larger proportion of the idiots than of the lunatics are of the independent or self-sustaining classes; 61 per cent. of idiots, and only 42 per cent. of the lunatics, are supported by their friends or their own estates." And the "idiots bear a much larger proportion to the lunatics among the natives than among the foreigners, being in the ratio of fifty-one native and seven foreign idiots for one hundred lunatics in each class respectively."

The commission was composed of Levi Lincoln, Increase Sumner and Edward Jarvis; Messrs. Lincoln and Sumner state, that the report is chiefly the work of their colleague, Doctor Jarvis, a gentleman already eminently known to the profession.

---

*The Action of Medicines in the System; or, On the Mode in which Therapeutic Agents Introduced into the Stomach produce their peculiar Effects on the Animal Economy.* By Fredk. Wm. Headland, M. B., B. A., F. L. S., &c. *Second American from the Second and Enlarged London Edition.* Philadelphia: Lindsay & Blakiston. 1856.

We are much pleased to see another edition of this truly valuable and suggestive work, as the very important though complex subject of which it treats, "the action of medicine in the system," needs all the light which modern science can throw upon it. The preface states, that "The first and second chapters have been left unaltered, but some new articles will be found scattered through the third and fourth chapters, and some additional observations and experiments recorded in the same.

No medical library should be without it.

*How to Nurse Sick Children.* New York: S. S. & W. Wood, 261 Pearl street.

We received the above little work several months since, and read it at the time with much pleasure. Its author, whoever he may be, wields an eloquent and forcible pen; while the kindness of feeling, which there can be no doubt chiefly prompted its writing, and the sound judgment displayed in all its directions, deserve the warm thanks of all engaged either in the attendance upon or treatment of sick children.

We venture to say, that no one will commence its perusal who will not both read it through and be much pleased with it.

